# Engineering Multiple Abiotic Stress Tolerance in Canola, *Brassica napus*

**DOI:** 10.3389/fpls.2020.00003

**Published:** 2020-02-25

**Authors:** Neeta Lohani, Divya Jain, Mohan B. Singh, Prem L. Bhalla

**Affiliations:** Plant Molecular Biology and Biotechnology Laboratory, Faculty of Veterinary and Agricultural Sciences, The University of Melbourne, Melbourne, VIC, Australia

**Keywords:** abiotic stress, *Brassica napus*, canola, multiple stress tolerance, CRISPR-Cas9, biotechnology, synthetic biology

## Abstract

Impacts of climate change like global warming, drought, flooding, and other extreme events are posing severe challenges to global crop production. Contribution of *Brassica napus* towards the oilseed industry makes it an essential component of international trade and agroeconomics. Consequences from increasing occurrences of multiple abiotic stresses on this crop are leading to agroeconomic losses making it vital to endow *B. napus* crop with an ability to survive and maintain yield when faced with simultaneous exposure to multiple abiotic stresses. For an improved understanding of the stress sensing machinery, there is a need for analyzing regulatory pathways of multiple stress-responsive genes and other regulatory elements such as non-coding RNAs. However, our understanding of these pathways and their interactions in *B. napus* is far from complete. This review outlines the current knowledge of stress-responsive genes and their role in imparting multiple stress tolerance in *B. napus*. Analysis of network cross-talk through omics data mining is now making it possible to unravel the underlying complexity required for stress sensing and signaling in plants. Novel biotechnological approaches such as transgene-free genome editing and utilization of nanoparticles as gene delivery tools are also discussed. These can contribute to providing solutions for developing climate change resilient *B. napus* varieties with reduced regulatory limitations. The potential ability of synthetic biology to engineer and modify networks through fine-tuning of stress regulatory elements for plant responses to stress adaption is also highlighted.

## Introduction

Plants as sessile organisms frequently face environmental conditions hostile to their growth and development. These disruptive environmental conditions are in the form of various abiotic and biotic stresses. Abiotic stresses have been predicted to limit global crop production by almost 70% (Boyer, 1982). Climate change has increased the frequency of adverse events, with simultaneous occurrence of multiple abiotic stresses leading to exacerbated negative impacts. Abiotic stresses include heat, cold, drought, salinity, waterlogging, heavy metal toxicity, nutrient deficiency, and oxidative stresses. Among these, drought, salt, and temperature stresses affect the geographical distribution and limit crop productivity. Approximately 40% and 7% of the global land area is affected by drought and salinity, making them the major environmental factors affecting crop productivity (Trenberth et al., 2014). The susceptibility of plants to abiotic stresses also varies with factors such as its timing, duration, and intensity of stress. Exposure to abiotic stresses leads to the alteration of numerous physiological, biochemical, and molecular processes in the plant. Under field conditions, plants encounter multiple stresses at once, making it challenging to develop stress-tolerant plants.

The global food market is composed of three main crop groups; cereals, oil crops, and legumes. Oil crops ranks second in world crop production after cereals and are of high economic worth for agriculture and trade worldwide. Among oil crops, *Brassica napus* (oilseed rape/rapeseed/canola) ranks second worldwide with an annual value of 41 billion U.S. dollars collectively (USDA, 2018).

*B. napus* is an amphidiploid formed by hybridization of diploid progenitor species, *B. rapa* and *B. oleracea* (Wang et al., 2011). Rapeseed/canola is versatile in its uses with the extracted oil being used for cooking, biofuel production, and in the ole-chemical and pharmaceutical industries with the meal after oil extraction used as a high protein animal feed (Friedt and Snowdon, 2009). Other than rapeseed/canola, *B. napus* also encompasses subspecies which produce tuberous (*B. napus* subsp. *Rapifera,* rutabaga) and leafy vegetables (*B. napus* subsp. *Pabularia,* leaf rape). Grown in the temperate climates of both northern and southern hemispheres, it is cultivated in different seasons (annuals or biennials) (Shahzadi et al., 2015; Zhu et al., 2016). Just like other temperate field crops, *B. napus* is also susceptible to multiple abiotic stresses (Elferjani and Soolanayakanahally, 2018). Drought, salinity, extreme temperatures, and cadmium toxicity are the most prevalent abiotic stresses affecting the growth and development of *B. napus*.

This review summarizes the current knowledge of abiotic stress responses in *B. napus* with an emphasis on possible biotechnological and synthetic biology routes for the development of climate-resilient varieties. We will first discuss the physiological impact of abiotic stresses followed by the fundamental question of how plants sense and adapt to these disruptive environmental conditions, with a further focus on the stress sensing and signaling cascades in *B. napus* along with other related crops. Multiple stress-responsive genes and studies focusing on developing abiotic stress-tolerant *B. napus* will be highlighted. The role of non-coding RNAs in response to stress conditions will also be discussed.

## Physiological Impact of Abiotic Stress in Canola

Different abiotic stresses adversely affect major biological processes in plants such as photosynthesis, stomatal conduction, rate of transpiration, protein synthesis, and metabolite accumulation (Zhang X. et al., 2014; Zhu et al., 2016; Elferjani and Soolanayakanahally, 2018). Abiotic stresses and their physiological consequences on plants are both shared and unique (Chinnusamy et al., 2004). For example, both drought and salt stress limit plant growth through somewhat different modes of action. Drought has been shown to lead to a decline in photosynthesis, whereas salt stress limits growth through enhanced ion toxicity (Chinnusamy et al., 2004). Drought or scarcity of water is one of the leading abiotic stresses imposed on plants in the current climatic scenario. In *B. napus* decrease in plant biomass, reduced chlorophyll content due to loss of pigments and damaged thylakoid membranes, reduced seed oil, and protein content are some of the physiological changes observed under drought (Rizwan et al., 2019). Relative water content (RWC) is a physiological measure of cellular hydration in the plants. Up to 48% of RWC loss in *B. napus* leaves been reported under drought conditions (Sabagh et al., 2019). However, drought is not the only abiotic stress that leads to cellular dehydration as it is also induced under salt stress, which causes a state of toxicity and osmotic stress (Zhang X. et al., 2014; Rezayian et al., 2018).

Amphidiploid *B. napus* is more tolerant to salinity in comparison to other diploid Brassica species suggesting an interspecific variation for salt tolerance (Purty et al., 2008). Still, the implications of salt stress on *B. napus* productivity are manifold due to Reactive Oxygen Species (ROS) mediated cell injury, reduced uptake of essential nutrients such as nitrogen, potassium, and decreased total fatty acid content (Rizwan et al., 2019). Similarly, under cadmium stress, *B. napus* has been reported to have altered fatty acid content in seeds along with the reduced activity of ROS mitigating antioxidant enzymes (Ding et al., 2018). Cadmium, a non-essential metal ion, when accumulated in high concentration, leads to cytotoxicity (Chmielowska-Bąk et al., 2014). Decreased photosynthesis efficiency due to declined chlorophyll content, reduced root growth, and shoot biomass are some adverse physiological features affected by cadmium (Benáková et al., 2017).

Temperature is a determining factor in plant productivity, which acts as a double-edged sword, extreme fluctuations on either end results in stress conditions. In the case of *B. napus,* 29.5°C was observed as a threshold and increase in temperature beyond this imposed constraint on the plant reproduction and yield (Morrison and Stewart, 2002). Reproduction is a plant developmental process most vulnerable to heat stress (Angadi et al., 2000; Lohani et al., 2019b). Following exposure of *B. napus* to heat stress at 35°C, reduction in pollen viability, germinability, fruit abortion, and reduced seed production were observed (Young et al., 2004). High-temperature results in a higher unsaturation ratio (oleic/linoleic acid ratio) of fatty acids leading to decreased oil quality (Gibson and Mullen, 1996; Aksouh-Harradj et al., 2006). The temperature has been reported to modify the C-N metabolism and gaseous exchange favoring protein accumulation at the expense of oil and carbohydrates (Canvin, 1965; Wang and Liu, 2014). In *B. napus,* exposure to extremely low temperatures has also been shown to reduce photosynthetic efficiency and induce membrane damage leading to electrolyte leakage (Megha et al., 2018a). Elferjani and Soolanayakanahally (2018) reported divergent responses of *B. napus* to heat, drought, and combination of both stresses in terms of seed yield and oil quality. While drought led to reduction in the carbon assimilation rate due to limitation of stomatal CO_2_ diffusion, the heat stress largely affected reproductive processes leading to significant reduction in the number of siliques and seed yield.

**Figure 1:**
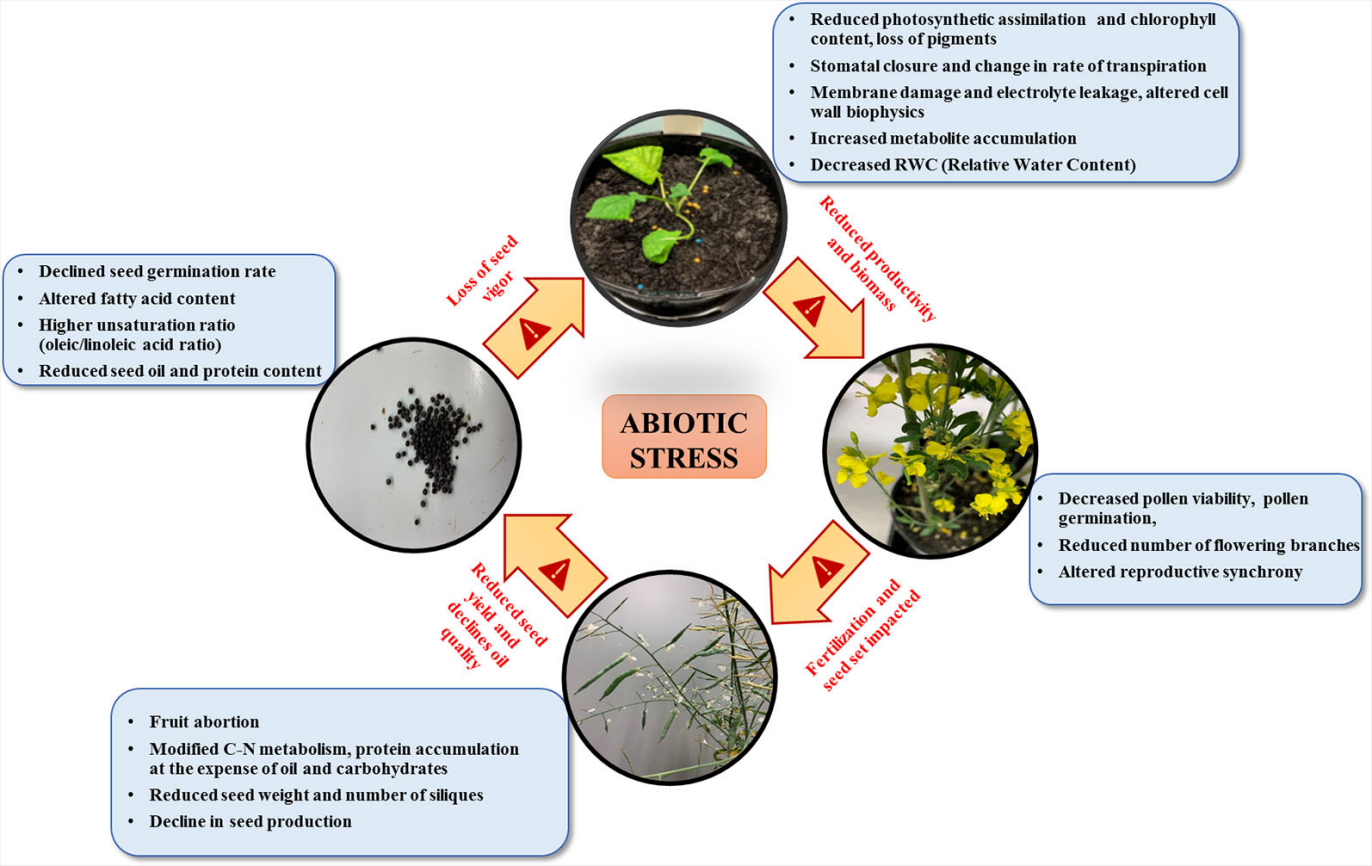
Physiological impact of multiple abiotic stresses in canola on different developmental stages.

**Figure 1** outlines the physiological impacts of various abiotic stresses on growth, development, and yield in *B. napus*. The physiological responses of plants to primary stresses such as salt, drought, and temperature are often interconnected, leading to secondary stresses such as osmotic and oxidative stresses (Wang et al., 2003). This interconnectivity requires compartmental cross-talk in plants for stress adaptation when facing multiple stresses simultaneously. For instance, drought-induced changes in cell wall's biophysical properties, temperature stress-induced fluctuations in membrane fluidity, and membrane damage under salt and cadmium stress, cumulatively contribute to the downstream stress signaling cascade. This highlights the intricate and elaborate nature of stress sensing, signaling, and response machinery that evolved in plants to endure abiotic stress conditions.

## Stress Sensing and Signaling

**Figure 2:**
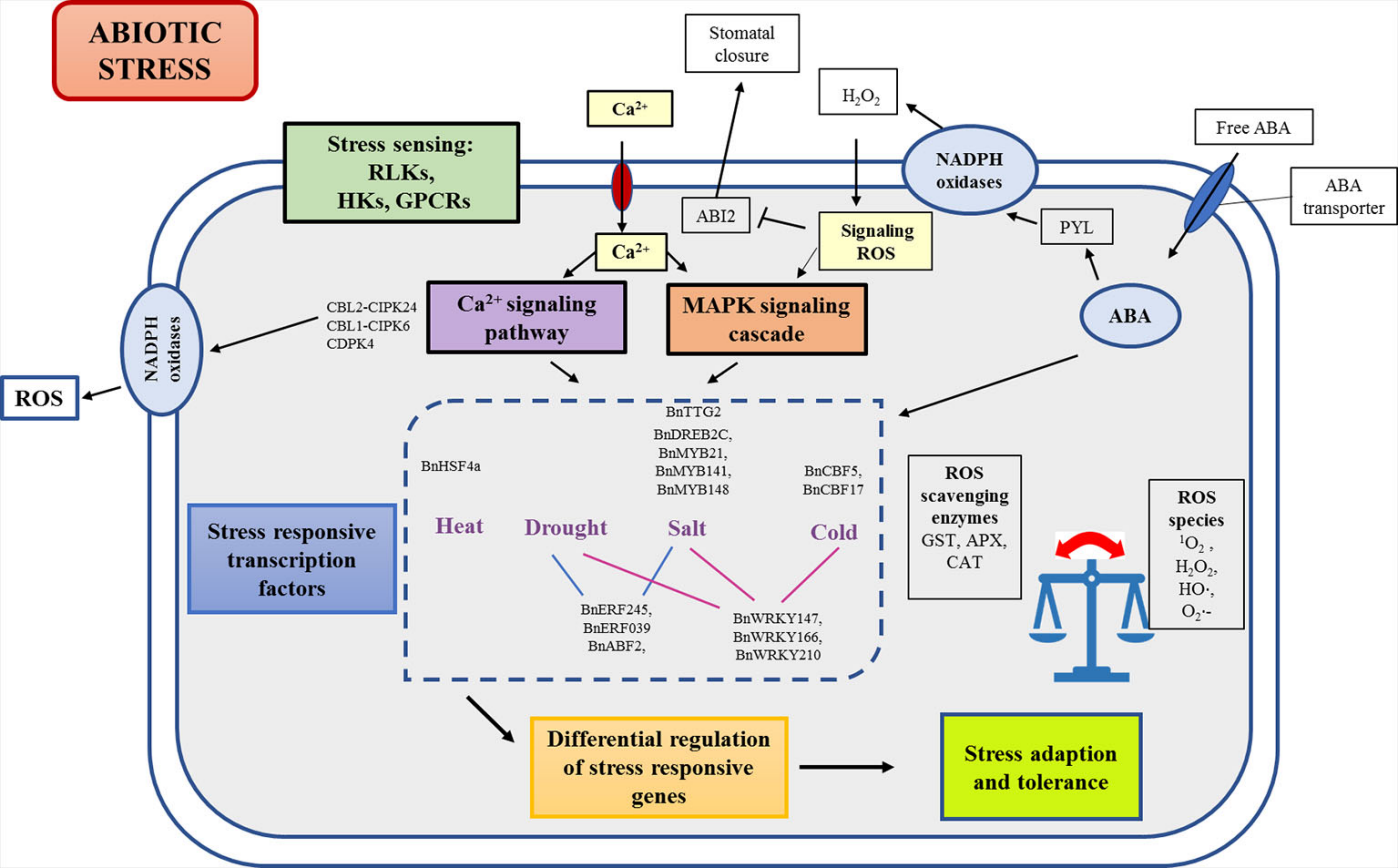
Schematic representation of abiotic stress sensing and signaling in *B. napus*. Initial incidence of stress sensing through receptors cascades the downstream stress response through secondary messengers such as calcium and ROS. Signal amplification and transduction through CDPK and MAPK signaling cascades leads to differential transcriptional regulation of stress responsive genes. ABA is involved not only in regulation of ABA responsive transcription factors to bring about stress adaption but also interacts with PYL family to coordinate guard cell shrinkage and stomatal closure leading to reduced transpiration. ABI2, ABA insensitive 2; APX, Ascorbate peroxidase; CAT, Catalase; CPKs, Calcium-dependent protein kinases; H_2_O_2_, Hydrogen peroxide; ^•^OH, hydroxyl radical; GPCRs, G-protein coupled receptors; GST, Glutathione S-transferases; HKs, Histidine kinases; MAPK, Mitogen activated protein kinases; PYL, Pyrabactin resistance like family; RLK, Receptor like kinases; ROS, Reactive oxygen species; ^1^O_2_, Singlet oxygen; O_2_^•^⁻, superoxide radical.

Acclimatization to stress is a complex process that involves a fine-tuned combination of genes, proteins, metabolites, and multiple regulatory pathways. The first step in abiotic stress response is sensing or perception of stress by plants. Sensors can be defined as a molecule or a structure that undergoes structural changes or transient loss of function, initiating a signaling cascade that leads to a response (Ruelland and Zachowski, 2010). These sensors cause reversible physical changes such as the change in membrane fluidity, protein conformational changes, partial separation, or melting of DNA and RNA strands. These changes further set in differential transcription control and regulation of stress-responsive genes, which then eventually constitutes a physiological stress response (**Figure 2**).

### Initial Stress Perception: Calcium Sensors

Cell surface or the cell membrane is often the first site of stress sensing with external stimuli resulting in fluctuations of cytosolic calcium level. The involvement of calcium (Ca^2+^) in multiple physiological and developmental processes consolidates its role of being a major player in the stress signaling cross-talk (Knight, 1999; Whalley and Knight, 2013). Variations in the calcium signature can be attributed to the type of stress, site of stress incidence, exposure rate and intensity (Chinnusamy et al., 2004). One of the most common responses of stress perception is increased intracellular accumulation of free Ca^2+^. Under cold stress, plasma membrane rigidification, and increase in Ca^2+^ accumulation induce cytosolic calcium signatures leading to altered expression of cold-responsive genes (Miura and Furumoto, 2013). Similarly, within minutes of temperature rise, a conserved transient calcium influx is observed in several model plants such as *Arabidopsis*, tobacco, and moss *Physcomitrella* (Saidi et al., 2011). The role of Ca^2+^ ions in activation of heat shock factors (HSFs) leading to further expression of heat shock proteins (HSPs) is critical in heat stress response (Saidi et al., 2011). As a secondary stress messenger, Ca^2+^ relays the stress signals from cell surface receptors to effector proteins, and initiate downstream responses. Calcineurin B-like proteins (CBLs), calmodulin (CaMs), calmodulin-like proteins (CMLs), and Calcium Dependent Protein Kinases (CDPKs/CPKs) are sensor relays requiring interaction with other target proteins for their regulation.

#### Calmodulin and Calmodulin-Like Proteins

CaMs and CMLs are a family of Ca^2+^ sensors in plants containing helix-loop-helix EF-hand domains and regulate downstream targets based on Ca^2+^ fluctuations. In *Arabidopsis,* members of seven CAMs and 50 CMLs have been characterized to be involved in responses to cold, heat, osmotic, and ionic stress (McCormack et al., 2005). These bind to calmodulin-binding transcription factors (CAMTAs), also referred to as signal responsive (SR) proteins. *Arabidopsis* CAMTA1, CAMTA2, and CAMTA3 contribute to low temperature and freezing tolerance by activation of CBF (C-repeat/DRE binding factor) transcription factors (Doherty et al., 2009). In *B. napus,* Rahman et al. (2016) identified eighteen CAMTAs, maximum of any other plant species reported so far. Rahman et al. (2016) predicted functional homology between AtCAMTA3, BnCAMTA3A1 and BnCAMTA3C1. AtCAMTA3 has been previously suggested to play a role in enhancing plant tolerance to cold and heat stress (Liu et al., 2005; Virdi et al., 2015). Efforts to identify and characterize CaMs or CMLs in *B. napus* like other plant species will significantly assist in understanding the specific CAMTA interactions and stress response.

#### Calcineurin B-Like Proteins

Unlike CaMs, which target a variety of proteins, CBLs explicitly interact with CBL-interacting protein kinases (CIPKs) or SNF1-related protein kinases SnRK3 (Chen et al., 2012). The structural composition of CIPKs involves an N-terminal kinase catalytic domain, a junction domain that connects it to the highly variable C-terminal regulatory domain (Chaves-Sanjuan et al., 2014). The C-terminal regulatory domain consists of the FISL motif with a unique 24 amino acid stretch, which is essential for the CBL-CIPK binding (Albrecht et al., 2001). Ten CBLs and 25 CIPKs have been identified in *Arabidopsis* (Weinl and Kudla, 2009). Characterization of CBL and CIPK genes in *B. napus* by Yuan et al. (2014) revealed the presence of 7 CBLs and 23 CIPKs. Interaction studies of BnCBL1-BnCIPK6 protein were confirmed by bimolecular fluorescence complementation (BiFC) and its upregulation during salt stress, osmotic stress, and response to ABA suggested its possible role in salt stress tolerance and ABA signaling in *B. napus* (Chen et al., 2012).

Overexpression of *B. napus* CBL gene, *BnCBL4,* and its interaction with *BnCIPK24* was also shown to be responsible in rescuing *sos3-1 Arabidopsis* mutants and thus resulting in enhanced salt tolerance (Liu et al., 2015). Signal transduction of intracellular accumulation of Ca^2+^ ion through CBL/CIPK signaling during salt stress includes salt overlay sensitive pathway (SOS). There are three major components of the plant SOS pathway with SOS3 acting as a Ca^2+^ sensor, SOS2 encoding a serine/threonine kinase, and SOS1 encoding a plasma membrane Na+/H+ antiporter (Chinnusamy et al., 2004). Maintenance of ion homeostasis inside the cell during salt stress is critical for the plant salt stress tolerance. Under salt stress conditions for binding Ca^2+^, SOS3 encodes a protein with the N-myristoylation motif and three EF-hand domains and activates SOS2. The SOS3-SOS2 complex controls the expression and activity of SOS1 through direct phosphorylation (Gong et al., 2004).

#### Calcium Dependent Protein Kinases (CDPKs/CPKs)

The third components of the Ca^2+^ sensing machinery in plants are the CDPKs, which are sensor responders with the ability to self-modify the confirmation through enzymatic action (Chen et al., 2012). This makes CPKs unique in their dual functionality in calcium-sensing and then responding through downstream phosphorylation events against the stress condition cues. Immense overlap and cross-talk is observed in CPKs stress response. Against stresses such as drought, cold, salt, and heat, there are multiple CPKs essential for response to specific stress stimuli.

Zhang, H. F., et al (2014) identified 25 CPKs in *B. napus* and further analyzed their expression levels under various abiotic stresses. Their findings suggested BnCPK4's interaction with Protein phosphatase 2C (PP2C) to regulate ABA-responsive transcription factors such as ABF1 and ABF4/AREB2 for signaling during drought stress. Ca^2+^ sensing and signal transduction by BnCPK4 in *B. napus* and activation of bZIP TFs AREB3 and AB15 highlight their involvement in the regulation of ABA and drought stress signaling (Zhang H. F. et al., 2014). Similarly, Wang W., et al (2018) used a mating-based split ubiquitin system (mbSUS) and BiFC to study the BnCPK2 interacting partners. They suggested the role of BnCPK2 in the regulation of ROS and cell death and to have possible interactions with NADPH oxidase-like respiratory burst oxidase homolog D (RbohD). Similar findings have been reported in which the majority of the CPKs are shown to modulate ABA signaling and ROS homeostasis in plant cells (Asano et al., 2012).

### G Protein-Coupled Receptors (GPCRs)

GPCRs are a class of stress receptors operating in plants that perceive the stress signal. GPCRs bind to various ligands, which relay information regarding the extracellular stress stimuli. Ligand binding to GPCRs induces conformational changes and facilitates the exchange of GTP for GDP, which then activates heterotrimeric guanine-nucleotide-binding proteins (G proteins). The activated GTP-bound Gα and Gβγ complexes then further bind to downstream cellular effectors. Signal termination requires hydrolysis of GTP to GDP by Gα subunit and reconfiguration into the inactive form (Chakravorty and Assmann, 2018). Coupling of ligand-bound G proteins with GPCRs is responsible for the activation of Ras-related small GTP-binding proteins, which in turn sets in Ca^2+^ mediated inositol triphosphate (IP3) signaling pathway in response to abiotic stress in canola (Shokri-Gharelo and Noparvar, 2018). In *B. napus*, heterologous overexpression of an inositol phosphate kinase homolog from *Thellungiella halophila* (*ThIPK2*) conferred resistance to salt, dehydration, and oxidative stresses (Zhu et al., 2009). Possible mechanism of action of *ThIPK2* in transgenic *B. napus* plants was a higher accumulation of Na^+^ ions in roots, higher proline content, and differential expression of stress-responsive genes.

In *Arabidopsis* GPCR2, the receptor for phytohormone ABA was also reported to be a GPCR. Located in the guard cell, it mediates the stomatal movement and thereby transpiration rate in response to ABA accumulation under stress (Tuteja and Sopory, 2008). GPCRs are also reported to control many cellular processes by regulating phospholipid signaling pathways (Tuteja and Sopory, 2008). In *B. napus*, expression of Gα, Gβ, and Gγ subunit encoding genes under different stress conditions has been examined. BnGA1, BnGB1, and BnGG2 genes encoding Gα, Gβ, and Gγ subunits respectively showed upregulation under salt and drought stress and downregulation in heat and cold stress (Gao et al., 2010a; Gao et al., 2010b; Gao et al., 2010c). All these genes also exhibited a common ABA regulated induction, indicating a possible role in the hormone signaling pathway. Rate of Gα protein-mediated GTP hydrolysis is fast-tracked by the regulator of G-protein signaling proteins (RGS); hence, they act as negative regulators of G-protein signaling. In *B. napus,* BnRGS1 was shown to interact with BnGA1. Its upregulation during PEG treatment, which simulates conditions similar to salt and drought stress, indicated a possible mode of action in an ABA-mediated manner during stress conditions (Chen et al., 2014).

### Receptor-Like Kinases (RLKs) and Histidine Kinases (HKs)

RLKs make up the largest gene family in plants with structurally similar proteins having an extracellular ligand-binding domain (ECLB), a single membrane-spanning transmembrane domain (TM), and an intracellular protein kinase catalytic domain (PKC) (Goff et al., 2007; Ye et al., 2017). The intracellular kinase domain plays the central role in signal transduction, which upon binding of a ligand to ECLB, modifies the protein conformation. Various RLK subfamilies such as proline-rich extensin like receptor kinases (PERKs), S-domain containing RLKs, lectin-like RLKs, and wall-associated kinases (WAKs) have been shown to play a role in abiotic stress response (Nongpiur et al., 2019). ABA-mediated abiotic stress response of RLKs has been documented in various crops such as *Arabidopsis*, rice, and *Glycine soja*. Many of these have also been characterized for tolerance against specific abiotic stresses such as GsCBLRK in salt stress and AtCRLK1 in cold and salt stress (Virdi et al., 2015). While analyzing the cross-talk and specificity between signaling mechanisms of salt and drought stress in *B. napus,* Luo et al. (2015) identified two drought stress-responsive RLKs along with glycosylphosphatidylinositol (GPI)-anchored salt receptor protein.

HKs are another class of receptors known to play a role in abiotic stress sensing. Membrane-bound HKs are known for their two-component system (TCS) of functioning. TCS for HK mediated osmosensing is already well established in bacteria and yeast. The system consists of a sensory histidine kinase (HIK) and a response regulator (RR) (Nongpiur et al., 2019). In plants presence of a His-containing phosphotransfer (Hpt) protein connects the initial sensory HIK to the ultimate RR while mediating as a signaling module. This permits for a multiple-step phosphorylation relay with the benefit of regulation through checkpoints for cross-talk and even negative regulation by specific phosphatases. The eventual modification of gene expression for stress response is mediated by the protein-protein and protein-DNA interaction of RR's effector domain. Light perception, cytokinin, and ethylene signaling all involve members of the two-component system (Singh et al., 2015). Previously, characterization of *Arabidopsis* histidine kinases AHK2, AHK3, and AHK4/CRE1 suggested their role as cytokinin receptors and negative regulators in ABA, drought and high salinity stress signaling (Tran et al., 2010; Kumar and Verslues, 2015). In *B. napus*, identification and functional characterization of five *B. napus* histidine kinases (BnCHK1–BnCHK5) revealed BnCHK1-BnCHK4 to be an AHK2 homolog and BnCHK5 as an AHK3 homolog respectively (Kuderova et al., 2015). These findings point towards a probable similar conserved mechanism of histidine kinases in *B. napus* through cytokinin signaling for developmental and stress response regulation.

Amplification and further transduction of the stress signals downstream of RLKs and HKs are carried out by an intricate cascade of various protein kinases.

### Mitogen-Activated Protein Kinase (MAPKs) Signaling Cascade

MAPK signaling cascade integrates and channels signal transduction for the expression of stress-responsive genes mediated through phosphorylation. Components of MAPK signaling cascade are involved in and act as converging points for multiple abiotic stress tolerance mechanisms (Chinnusamy et al., 2004).

MAPK signaling cascades comprises of MAPKK kinases (MAPKKKs, MAP3K, or MEKK), MAPK kinases (MAPKKs, MAP2Ks, MKKs, or MEKs), and MAPKs (MPKs). MAP kinases through phosphorylation function as on-off signaling switches aiming downstream targets. Successive phosphorylation/dephosphorylation of serine or threonine residues by MAPKKKs dictates activation of MKKs and then threonine and tyrosine residues for activating MPKs (Sun et al., 2014). The activated terminal MAPKs then proceed forward with the signal transduction by phosphorylation mediated control of transcription factors or enzymes. In *Arabidopsis,* a total of 80 MAPKKK, 10 MKK, and 20 MPK genes have been identified (Liang et al., 2013). A lot more MKK and MPK genes have been functionally characterized compared to MAPKKK genes, even though they constitute the most abundant family out of the three. The *Arabidopsis* flagellin cascade (AtFLS2-AtMEKK1, AtMKK4/AtMKK5, AtMPK3/AtMPK6, AtWRKY22/AtWRKY29) working in defence response was one of the first complete MAPK signaling cascades to be characterized in plants (Asai et al., 2002). There is a noticeable gap in the comprehensive understanding and characterization of MAPK signaling cascade routes with their target transcription factors and stress-responsive genes in *B. napus*. Working towards this goal, Liang et al. (2013) identified 7 MKK and 12 MPK members. Combination of Yeast two-hybrid (Y2H) interaction studies and BiFC assay helped in identifying possible interactions in the MAPK signaling cascade and TFs such as BnMKK9-BnMPK5/9/19/20, BnMKK9-BnMPK1/2-BnWRKY53, and BnMKK2/4/5-BnMPK3/6-BnWRKY20/26. Further, Sun et al. (2014) identified 66 MAPKKK genes in *B. napus*. Expression of *BnMAPKKK* genes was regulated by hormone-induced stress stimuli and other abiotic stresses, including cold, heat, and oxidative stress. They also reported the role of MAPKKK18 and 19 in eliciting ROS accumulation and hypersensitive response (HR) like cell death upon transient expression in tobacco leaves *via* a possible interaction with BnMKK9. Its interaction with the previously established MKK9–BnMAPK1/2–BnWRKY53 cascade needs further investigation. BnMAPKKK4 was also reported to cause a similar ROS mediated response upon transient overexpression in tobacco leaves (Li L. et al., 2015). Possible interaction between BnMAPKKK4 and MAPK3 was highlighted in eliciting the ROS response.

### ABA-Dependent Abiotic Stress Signaling

Commonly known as the stress hormone, ABA, and its role in plant stress response is highlighted in the ABA-dependent signaling pathway. The ABA signaling pathway, mainly functioning in the plasma membrane, plays a vital role in the plant's response to salt, cold, hypoxia, and drought stress. Ca^2+^ and protein kinase-mediated sensing of stress leads to activation of SnRK2, which sets in ABA accumulation. This increased ABA accumulation under stress conditions is identified by protein receptors of the pyrabactin resistance like (PYL) family (Vishwakarma et al., 2017). ABA then forms a tri complex with 2C protein phosphatases (PPC2), which almost acts as co-receptors leading to the increased binding affinity of ABA and PYL.

*Cis*-acting element, ABA-responsive element (ABRE), and transcription factors, ABRE-binding protein/ABRE-binding factors (AREB/ABFs) regulate the ABA-dependent gene expression (Yoshida et al., 2014). Varied targets in stress response mediate the physiological modifications such as stomatal closure, flowering time control, chromatin regulation, and RNA splicing. High levels of endogenous ABA content in drought-tolerant cultivars compared to drought susceptible cultivars have been reported in barley (Zhang X. et al., 2014), indicating an inherent requirement of higher ABA levels for stress-related response and improved tolerance. Exogenous application of ABA also induces a stress response in plants enhancing their adaption ability, thus indicating the importance of its action under stress conditions (Sah et al., 2016).

ABA is also reported to be involved in seed desiccation tolerance and regulating various aspects of seed development and dormancy (Chinnusamy et al., 2004). The role of *B. napus* BnABI3, a B3 domain-containing protein known for functioning in the ABA signaling pathway was investigated by Xu and Cai (2019) during seed development in *Arabidopsis*. They reported direct involvement of BnABI3 in seed coat development and desiccation tolerance. Their findings also suggested the possible role of BnABI3 in the coordination of flowering time and response to cold stress as its overexpression led to delayed flowering and rescue of the cold-induced green seed phenotype. Similarly, upregulation of BnABI5, a basic leucine zipper transcription factor responsible for the regulation of multiple LEA genes, was observed under both ABA and PEG induced water stress for enhanced seed dormancy in *B. napus* (Li et al., 2005).

Shrinking of guard cells in response to drought to prevent loss of water through transpiration is an important stress adaptive response. Proteomic analysis of *B. napus* guard cell protoplasts after ABA treatment showed upregulation of 66 proteins. The majority of which is involved in photosynthesis and stress responses also showed an overlap with the drought inducible proteins. These observations presented an insight into the physiological changes manifesting out of the ABA signaling pathway, such as reorganization of the cytoskeleton and ROS homeostasis (Zhu et al., 2016). Zhu and Assmann (2017) used exogenous application of ABA hormone for differential metabolomic profiling in *B. napus* guard cells. They identified 29 primary and 48 secondary ABA-responsive metabolites possibly functioning in this intricate intracellular signaling in guard cell response to drought.

### Other Phytohormones Involved in Abiotic Stress Signaling

Although among phytohormones, the principal regulator of abiotic stress response is ABA, increasing evidence highlight the involvement of other phytohormones such as salicylic acid, jasmonates, ethylene, brassinosteroids, auxins, cytokinins, and gibberellins as well. The nature of phytohormone mediated regulation is further complex as they can act either directly or orchestrate abiotic stress response *via* cross talking networks involving other phytohormones, MAP Kinases, ROS, sugar, and other secondary messengers (Smékalová et al., 2014; Ljung et al., 2015). The role of these phytohormones in positively or negatively regulating abiotic stress tolerance has been extensively reviewed (Peleg and Blumwald, 2011; Wani et al., 2016).

Complete understanding of the interaction of phytohormones and stress signaling is lacking, however, in the light of recent investigations, this interaction is becoming very evident. For instance, ethylene signaling may cause repression of CBF pathway in Arabidopsis, thus negatively regulating cold tolerance (Shi et al., 2012). Ethylene signaling also negatively regulates salt and drought tolerance. Similarly, a reduced level of GA has been reported to possibly restrict plant growth in response to several abiotic stresses and increased levels promote growth and assists plant escape during shade or submergence (Bailey-Serres and Voesenek, 2010). Jasmonate biosynthesis and signaling pathway can positively regulate various abiotic stress responses (Dar et al., 2015). Regulation of stress responsive transcriptional machinery by cytokinins and its cross-talk with stress signaling has also been suggested (Zwack and Rashotte, 2015). Possible association of GA signaling with JA signaling *via* DELLA as well as JA and ethylene signaling further validates the complex cross-talk network (Colebrook et al., 2014; Kazan, 2015).

The exogenous application of phytohormones is well documented to mitigate the negative implications of abiotic stress in canola (Kurepin et al., 2008; Farhoudi and Saeedipour, 2011; Alam et al., 2014; Hasanuzzaman et al., 2014). SA application imparted salt stress tolerance in B. napus by improving the performance of the antioxidant enzymes such as GR, GST, GPX, CAT, thereby reducing the extent of oxidative damage (Hasanuzzaman et al., 2014). Pretreatment of B. napus seedlings with SA alleviated drought induced symptoms by antagonistically interacting with ABA. It also enhanced the expression proline synthesis related and redox reducing genes. Also, SA possibly fosters drought tolerance in *B. napus* by mediating transcriptional regulation of sugar accumulation under drought stressed conditions. Lee et al. (2019a) reported reduced expression levels of *NCED3* (involved in ABA synthesis), *PDF1.2* (JA signaling gene) and Lee et al. (2019b) reported reduced expression of ABA-dependent sucrose signaling genes *SnRK2.2* and *AREB2* strongly indicating an averse relationship between SA and ABA in regulation drought stress response. Similarly, pretreatment of *B. napus* seedlings with brassinolide enhances thermotolerance by increasing the endogenous levels of ABA. 24-Epibrassinolide treatment also enhances tolerance against heat, cold, and drought (Kagale et al., 2007). Possible mode of action of BRs in abiotic stress is by regulation of stress responsive transcriptional machinery.

### Reactive Oxygen Species—A Major Player in Stress Response

**Figure 3:**
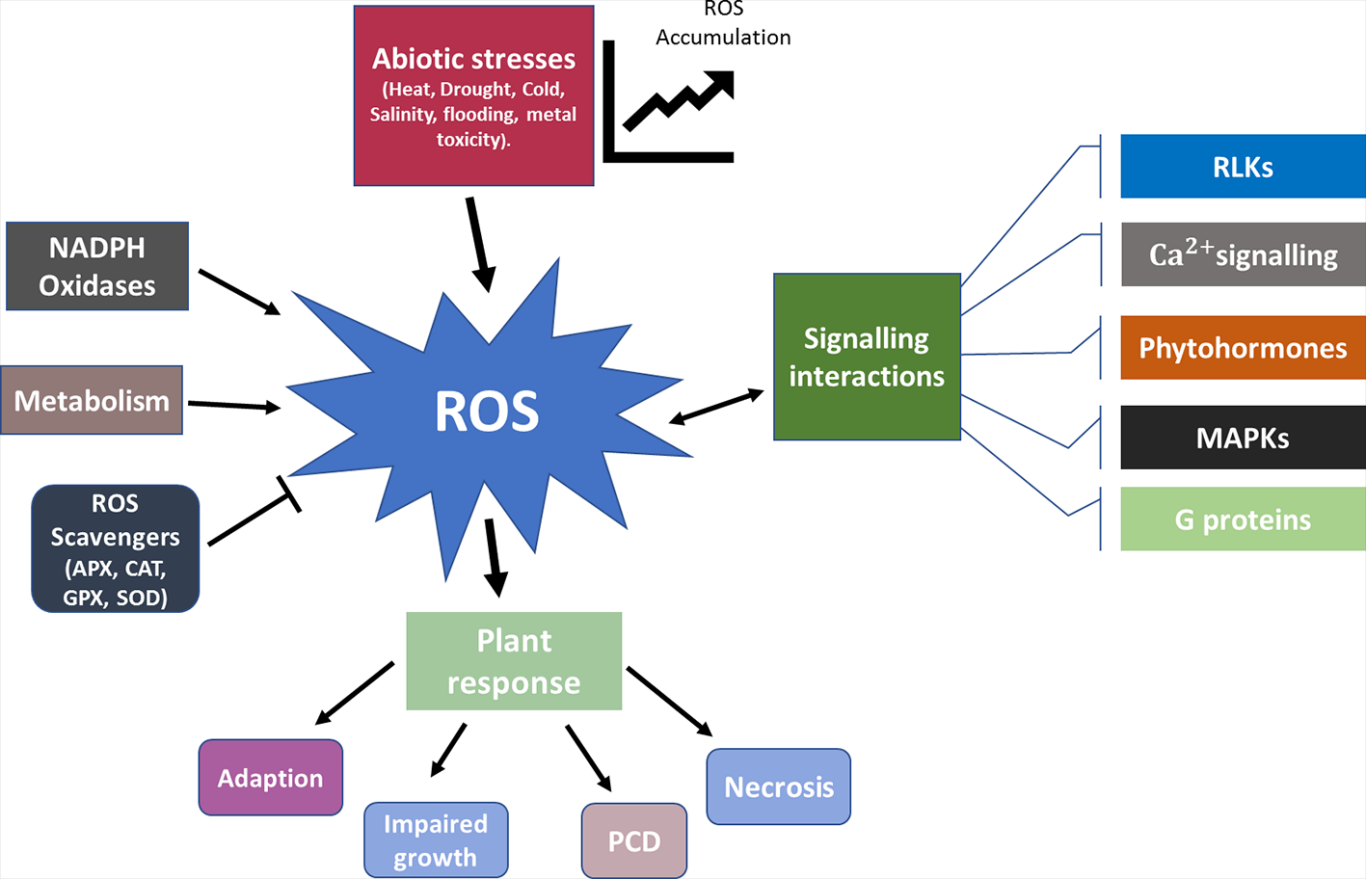
Schematic representation for functioning of ROS in plant stress response. During normal functioning of the plant metabolic processes ROS is produced and scavenged in a harmonious balance. Abiotic stresses skew the ROS concentration gradient and lead to an increased accumulation. ROS and its interaction with other signaling mechanisms such as RLKs, phytohormones, G proteins, MAPKs, and Ca^2+^ play a vital role in determining the subsequent response. Based on the severity of the stress the plant response ranges from adaption, impaired growth, PCD to necrosis. APX, Ascorbate peroxidase; CAT, Catalase; GPX, Glutathione peroxidase; MAPKs, Mitogen activated protein kinases; PCD, Programmed cell death; RLKs, Receptor like kinases; SOD, Sodium Dismutase.

Among all the biochemical responses to stress in plants, ROS plays a key role in acclimatization to abiotic stresses. ROS metabolism and the antioxidant defence system involved in abiotic stress response has been extensively reviewed (Gill et al., 2010; Suzuki et al., 2012; Petrov et al., 2015). Produced as a by-product of metabolic reactions in processes such as respiration, fatty acid oxidation, and photosynthesis, ROS helps maintain the ionic balance in various cellular components (Sharma et al., 2012). Contributing to the majority of growth and developmental stages of a plant, the ROS regulatory system implies processes including programmed cell death, autophagy, and response to stress (Mittler, 2017). Production of different forms of ROS, including singlet oxygen (^1^O_2_), hydrogen peroxide (H_2_O_2_), hydroxyl radical (HO·), and superoxide anion radical (O_2_·) is enhanced under stress (Sharma et al., 2012). This creates conditions that are highly damaging to biomolecules and sets in a cellular state known as oxidative stress. Increased ROS during stress also triggers transient or permanent protein modifications. These ROS induced post-translational modifications of proteins lead to conformational changes in enzymes, thereby altering transcriptional regulation and plant metabolic processes (Choudhury et al., 2017). **Figure 3** provides a schematic representation for functioning of ROS in plant stress response.

Different ROS signatures arising from abiotic stresses determine the specificity of the acclimation response (Choudhury et al., 2017). In plants NADPH oxidases, homologs to the respiratory burst oxidases (RBOHs) are involved in the ROS production network. They also play a role in the initiation and propagation of cell-to-cell systematic signal through H_2_O_2_ accumulation by generating a ROS wave. This acts as an overall signal to systemic tissues about the localized abiotic stress stimuli (Baxter et al., 2014). The *Arabidopsis* genome encodes 10 RBOH proteins (RBOHA-J) with varying functionality in plants response to different abiotic stresses (Torres and Dangl, 2005; Wang et al., 2013). AtRobhD and AtRobhF genes have been characterized to show ROS dependant ABA mediated signaling in guard cells and stomatal closure (Kwak et al., 2003). AtRBOHD assists AtRBOHF for ROS production to regulate Na^+^/K^+^ homeostasis under salt stress (Liu and He, 2016). The nine RBOH genes (OsRbohA-OsRbohI) identified in rice have been shown to have changes in expression patterns in response to various environmental stresses (You and Chan, 2015), with both OsRbohA and OsRbohC having increased abundance in drought stress (Wang et al., 2013). Even with their vital role in the ROS production network, in case of *B. napus*, there is still an existing gap in identification and characterization of RBOHs, which could potentially lead to better understanding of ROS mediated stress signaling. Production and scavenging of ROS balance dictates the maintenance of cellular homeostasis. This balance of production and scavenging is in harmony during unstressed conditions, but most environmental stresses trigger a disturbance with increased ROS production. As a guarding mechanism against this, plants are equipped with antioxidants of enzymatic and non-enzymatic nature. These include ROS scavenging enzymes such as superoxide dismutase (SOD), glutathione peroxidase (GPX), ascorbate peroxidase (APX), and catalase (CAT) along with compatible solutes such as proline (Pro) and glycinebetaine (GB) working to limit ROS induced injury. Increased activity of APX and glutathione S-transferase (GST) was observed in *B. napus* seedlings under cadmium stress (Hasanuzzaman et al., 2017). Similarly, Hyola varieties of canola showed a significantly high activity of APX and CAT under salt stress (Heidari, 2010). Comprised of ascorbate, glutathione, ascorbate peroxidase, monodehydroascorbate reductase, dehydroascorbate reductase, and glutathione reductase the ascorbate-glutathione (AsA-GSH) pathway plays a vital role in detoxifying ROS by improving osmoregulation, water use efficiency and nutrient status (Hasanuzzaman et al., 2019).

ROS also communicates with other signaling networks for stress adaption responses. Involvement of H_2_O_2_ towards ABA-mediated stomatal closure through inactivation of ABI2, a 2C protein phosphatase, and negative regulator of ABA (Pandhair et al., 2006). The heterologous overexpression of AtABI2 (group A PPC2 gene) in *B. napus* also resulted in transgenic *B. napus* lines with decreased drought tolerance, highlighting the negative regulation of ABA. ROS and its role in the maintenance of the cell's redox potential under stress are not only limited to interaction with ABA but also include other plant hormones such as gibberellins (GAs), Auxin, brassinosteroids (BRs), ethylene (Choudhury et al., 2017). ROS induces changes in level of endogenous plant hormones, leading to modification in their impact and activity (Lee et al., 2019a). Recent research in ROS signaling has greatly enhanced the understanding of this cross-talk between these signaling pathways, wherein ROS acts as key a regulator not only coordinating plant development but also response and adaption to stress.

## Multiple Abiotic Stress Responsive Genes in Canola

To bring about the required changes for stress adaption and response, the sensing and signaling cascade leads to the activation of molecular networks involved in the expression of specific stress-related genes and metabolites. The availability of the *B. napus* genome sequence in 2014 (Chalhoub et al., 2014) led to the identification and characterization of several stress-responsive gene families in *B. napus* (**Table 1**). Functional characterization of several abiotic stress-responsive *B. napus* genes has been carried out by ectopic expression in model plants *Arabidopsis* and tobacco (**Table 2**). An effective way to enhance crop adaptability or tolerance in multiple stress occurrences is to dissect the multifaceted stress-responsive regulatory networks as well as understand the specificity and cross-talk of those pathways leading to the identification of key genes.

**Table 1:** Abiotic stress responsive gene families identified in *B. napus*.

Gene Family	Stress-Responsiveness	Putative members	Reference
***Transcription Factors***						
**AP2/ERF**	Salt, cold	321	Owji et al. (2017)
**HSF** (Heat Stress Transcription Factors)	Heat, Drought, high CO_2_	64	Zhu et al. (2017)
**R2R3-MYB**	Salt, oxidative	249	Hajiebrahimi et al. (2017)
**WRKY**	Cold, salt, drought	287	He et al. (2016)
**NAC**	Cold, heat, Hormone treatment	60	Wang, B., et al (2015)
**bZIP**	NA	247	Zhou et al. (2017)
**NF-Y** (Nuclear factor-Y)	Salt, drought, ABA treatments	33	Xu et al. (2014)
**MADS-box**	Drought, heat, hormone treatment	307	Wu et al. (2018)
**WOX** (WUSCHEL-related homeobox)	Drought, salt	58	Wang, M. M., et al (2018); Li, M. D., et al (2019)
**ARF** (Auxin Response factor)	Hormone treatments	66	Wen et al. (2019)
**NLP** (NODULE-INCEPTION like)	Nitrogen deficiency	31	Liu, M., et al (2018)
**GRAS TFs**	Drought, salt	87	Li, M., et al (2019)
***Kinases***						
**MAPKKK** (Mitogen-activated protein kinase kinase kinase)	Hormone treatments	66	Sun et al. (2014)
**CPK** (Calcium dependent protein kinase)	Salt, cold, heat, drought, hormone treatment	25	Zhang, H. F. et al (2014)
**CBL** (Calcineurin B-like proteins) and **CIPK** (CBL-interacting protein kinases)	Salt, cold, heat, drought, hormone treatment	7 CBL, 23 CIPK	(Yuan et al., 2014)
**SnRK2** (Sucrose non-fermenting-1-related protein kinase 2)	Drought	30	Yoo et al. (2016)
***Transporters***						
**Aquaporins**	Boron deficiency	121	Yuan et al. (2017)
**ABC** (ATP Binding cassette) transporter	Cadmium	314	Zhang et al. (2018b)
**MTG**	Cadmium	270	Zhang et al. 2018a
**SUT/SUC**	Drought, salt, heat, hormone treatment	22	Jian et al., 2016a
**SWEET**	Drought, salt, heat, hormone treatment	68	Same
**HMA**	Cadmium	31	Li et al. (2018)
***Other gene families***						
**FAD** (Fatty acid desaturase)	Cadmium, salt	68	Xu et al. (2019)
**Dehydrins**	Cold		Maryan et al. (2019)
**Metallothionein**	Heavy metal	16	Abdelmigid (2016); Pan, Y., et al (2018)
**CKX** (Cytokinin oxidase/Dehydrogenase)	Drought, salt	23	Liu, P., et al (2018)
**PYL** (Pyrabactin resistance-1 like)	Drought, heat, salt	46	Di et al. (2018)
**Family-1 UDP glycosyltransferase**	Multiple abiotic stresses	251	Rehman et al. (2018)
**SPX**	Phosphate deficiency	69	Du et al. (2017)
**Galactinol synthase**	Hormone treatments	20	Fan et al. (2017)
**Aux/IAA** (Auxin/indoleacetic acid) genes	NA	119	Li, H. T., et al (2017)
**LAC** (Laccase gene family)	Cadmium	45	Ping et al. (2019)
**GST** (Glutatione transferase)	Drought, Salt, Heavy metal	179	Wei et al. (2019)
**VOC** (Vicinal oxygen chelate proteins)	Drought	38	Liang et al. (2016)
**LEA** (Late embryogenesis abundant)		108	Liang et al. (2016)
**SAP** (Stress Associated proteins) with A20/AN1 Zinc finger motifs	Cold, Heat	16/57 SAP	He et al. (2019)

**Table 2:** Summary of functional characterization of putative stress responsive *B. napus* genes by overexpression studies in model plants.

Gene	Gene description	Target	Response	Reference
***CPK2***	Kinase	Tobacco	Regulation of ROS and cell death	Wang, W., et al (2018)
***MAPKKK 4***	Kinase	Tobacco	Regulation of ROS and cell death	Li, L., et al (2015)
***MKK1***	Kinase	Tobacco	Multiple stresses	Yu et al. (2014)
***MAPKKK18, 19***	Kinase	Tobacco	Regulation of ROS and cell death	Sun et al. (2014)
***NAC 19, 82***	NAC TFs	Tobacco leaves	Regulation of ROS and cell death	Wang, B., et al (2015)
***NAC87***	NAC TF	Tobacco and *B. napus* protoplasts	Regulation of ROS and cell death	Yan et al. (2017)
***NAC56***	NAC TF	Tobacco and *B. napus* protoplasts	Regulation of ROS and cell death	Chen et al. (2017)
***NAC55***	NAC TF	Tobacco and *B. napus* protoplasts	Regulation of ROS and cell death	Niu et al. (2016)
***NAC103***	NAC TF	Tobacco	Regulation of ROS and cell death	Niu et al. (2014)
***NAC485***	NAC TF	*Arabidopsis* and *B. napus*	Abiotic stress response	Ying et al. (2014)
***TTG2***	WRKY TF	*Arabidopsis*	Salt stress	Li, Q., et al (2015)
***HSFA4a***	HSF TF	*Arabidopsis*	Desiccation tolerance in seeds	Lang et al. (2017)
***CDF1***	Dof TF	*Arabidopsis*	Freezing tolerance	Xu and Dai (2016)
***ERF-2 like***	Ethylene response factor 2-like	*Arabidopsis*	Submergence and oxidative tolerance	Lv et al. (2016)
***ABF2***	bZIP TF	*Arabidopsis*	Drought and salt tolerance	Zhao, B. Y. et al (2016)
***NCED3***	Involved in ABA regulation	*Arabidopsis*	ABA, ROS and NO accumulation	Xu and Cai (2017)
***Pht1;4***	Phosphate transporter	*Arabidopsis*	Phosphate uptake	Ren et al. (2014)
***ABI1***	Protein phosphatase	*Arabidopsis*	Drought stress	Babula-Skowrońska et al. (2015)
***LEA4-1***	Late-embryogenesis abundant (LEA) proteins	*Arabidopsis*	Salt and drought	Dalal et al. (2009)

### Transcription Factors

In the plant genome, out of the total coding sequences present, 5% to 7% is utilized for the expression of TFs. WRKY, MYB (myeloblastosis), bZIP (basic leucine zipper), AP2/ERF (APETALA2/ethylene-responsive factors), HSF (Heat Shock Factors), and NAC are some of the most prominent families of stress-responsive TFs. Out of these, WRKY, NAC, and AP2/ERFs are unique to plants (Lan Thi Hoang et al., 2017). Genetically manipulating the expression of TFs for imparting or enhancing abiotic stress tolerance is a widespread approach as most of them are involved in early stress response, and they control the expression of stress-responsive genes.

#### AP2/ERF TFs

AP2/ERF TFs can integrate responses to various stress stimuli such as Ca^2+^, ROS, MAPKs, and SnRKs mediated phosphorylation, and partake in stress responsive networks. This TF superfamily is further divided into five subfamilies: DREB (Dehydration Responsive Binding Element), ERF, AP2, Soloist, and RAV (Related to ABI3 and VP1) (Lan Thi Hoang et al., 2017). Owji et al. (2017) identified 321 putative AP2/ERF TFs in *B. napus* and also suggested the potential role of BnERF245 and BnERF039 in drought and salt tolerance. Functional characterization of the BnERF-2 like gene from *B. napus* in *Arabidopsis* highlighted their role in regulating antioxidant machinery for enhancing abiotic stress tolerance (Lv et al., 2016). Members of the DREB subfamily are recognized for their induction during multiple abiotic stresses (Mizoi et al., 2012). Salt stress-induced expression of DREB2C in Arabidopsis and its role in inducing salt stress tolerance by regulation of stress-responsive genes (RD29A, RD29B, and COR15A) upon overexpression was reported by Song et al. (2014). They subsequently overexpressed AtDREB2C in *B. napus* conferring improved salt tolerance due to higher accumulation of Na+, higher retention of water, better growth, and lower relative water content as compared to wild type.

CBF/DREB1s interact specifically with CRT/DRE cis-elements to direct the transcription of COR (cold regulated) genes. Transgenic *B. napus* lines overexpressing Arabidopsis *CBF* genes (*CBF1/DREB1b, CBF2/DREB1c,* and *CBF3/DREB1a*) showed enhanced freezing tolerance due to induction of CBF-targeted orthologous *B. napus* gene *Bn115* (Jaglo et al., 2001). Similarly, homologous overexpression of two *BnCBF/DREB1*-like genes (*BnCBF5* and *BnCBF17*) enhanced freezing tolerance in transgenic *B. napus* plants (Savitch et al., 2005). BnCBF17 overexpressing plants performed better in comparison to the BnCBF5 overexpressing canola, probably due to higher *cor* genes expression. Overexpression of these genes also partially regulated the expression of genes involved in chloroplast photosynthetic development, photosynthesis, Calvin cycle, starch, and sucrose biosynthesis, thus enhancing the photosynthetic efficiency in response to cold stress. Other important gene candidates from this superfamily are yet to be investigated in *B. napus*. However, the role of some of AP2/ERF TFs in imparting stress tolerance in other crops has been reported. For instance, overexpression of ERF1 imparts salt tolerance and freezing tolerance in rice and wheat, respectively (Schmidt et al., 2013; Zhu et al., 2014). Similarly, DREB sourced from various crops such as ZmDREB2A in maize (Qin et al., 2007), OsDREB1s and OsDREB2s in rice (Matsukura et al., 2010) and TaDREB1 in wheat (Shen et al., 2003), when overexpressed in plants enhanced abiotic stress tolerance. Further understanding of abiotic stress responses and the involvement of AP2/ERF TFs in canola is warranted.

#### HSFs

HSFs play a crucial role, not only in heat stress response, but other abiotic stresses as well (Guo et al., 2016). HSFs have a conserved DNA-binding domain (DBD) which recognizes heat stress elements (HSE) along with other cis-elements such as STRE (stress-responsive element), DRE/CRT (drought/cold-responsive element), and MYCATRD22 (dehydration, ABA-responsive element) in promoter sequences of the genes they regulate. Zhu et al. (2017) identified 64 *Hsf* encoding genes in *B. napus,* making it the largest *Hsf* gene family in eudicots so far. The role of hybridization and allopolyploidization in shaping the structure of the *Hsf* gene family has been reported (Lohani et al., 2019a). Most *Bn*HSFs were induced under heat as well as drought stress suggesting their role in multiple abiotic stress acclimatization in canola (Zhu et al., 2017). Lang et al. (2017) functionally characterized BnHSF4a in *Arabidopsis* and highlighted its role in desiccation tolerance of seeds by upregulation of genes *GolS1*, *GolS2*, and *raffinose synthase 2 (BnRS2)* which are involved in osmoregulation in plant cells. Ectopic overexpression of *Hsf* genes has been reported to enhance, salt, drought, and thermotolerance in other crops. For instance, overexpression of *GmHSFa1* in soybean (Zhu et al., 2006), *SlHSFA1,* and *SlHSFA3* in tomato and *TaHSFA6f* in wheat enhanced the thermotolerance of transgenic plants (Guo et al., 2016). Similarly, the silencing of *Hsf* genes resulted in the negative regulation of stress tolerance, e.g., *OsHSF4A* knockout rice plants showed decreased cadmium tolerance (Shim et al., 2009) and *SlHSFA2* knockout tomato lines were reported to have reduced reproductive thermotolerance (Fragkostefanakis et al., 2016).

#### WRKY TFs

WRKY TFs are yet another class of TFs playing a crucial role in multiple abiotic stress responses. They are characterized by the presence of the conserved motif WRKYGQK in the sixty amino acid long WRKY domain (Rushton et al., 2010). Phosphorylation induced signal transduction from MAPKKKs to MAPKs activates various substrates, including TFs from the WRKY family. He et al. (2016), through comparative transcriptome analysis, identified 287 WRKY TFs in *B. napus* and validated the multiple stress responsiveness of BnWRKY147, BnWRKY166, and BnWRKY210 under simultaneous low temperature, salinity, and drought stress. Li, Q., et al (2015) overexpressed *BnTTG2* (homolog of *Arabidopsis* AtWRKY44 TF) in *B. napus* and observed that *BnTTG2* is a transcriptional repressor under salt stress. The transgenic *B. napus* plants were hypersensitive to salt stress and showing lower expression of genes involved in IAA synthesis such as *TRP5* and *YUCCAA2*. Similarly, GmWRKY13 from soybean conferred increased salt sensitivity in transgenic *Arabidopsis* plants (Zhou et al., 2008). However, other WRKY factors such as OsWRKY45 and OsWRKY72 when overexpressed conferred enhanced salt and drought tolerance in transgenic *Arabidopsis* (Song et al., 2010). These studies highlight that the differential stress regulatory nature of different WRKY genes. It will be thus, worthwhile to explore the role of different *BnWRKY* genes in response to multiple stresses.

#### MYB TFs

MYB TFs function by specifically binding to MYB binding sites and are classified depending on the number of repeats in the MYB domain. Most extensively studied MYB TFs are the R2R3-type MYB proteins. Investigation of the R2R3-MYB gene family in *B. napus* by Hajiebrahimi et al. (2017) has led to the identification of 249 R2R3-MYB genes. Based on RNA-Seq data, *BnMYB21*, *BnMYB141*, and *BnMYB148* have been suggested as candidate genes that can be over-expressed to improve salt-tolerance in *B. napus*. The transgenic studies carried out in other crops highlight the importance of MYB genes in stress tolerance. Transgenic soybean overexpressing *AtMYB44* showed enhanced salt and drought tolerance (Seo et al., 2012). Similarly, overexpression of *LeAN2* (an anthocyanin associated R2R3-MYB TF) in tomato conferred thermotolerance along with higher anthocyanin accumulation (Meng et al., 2015). Functional characterization of MYB genes from different crops in model plants has also highlighted the positive regulation of abiotic stress response by these TFs.

#### NACs TFs

NACs TFs are plant-specific TFs with a highly conserved N-terminal NAC domain and a variable CT functioning activation domain. The role of NAC TFs in abiotic stress tolerance is well documented (Nakashima et al., 2012). Sixty NAC TFs have been identified in *B. napus* (Wang B. et al., 2015). A number of *BnNAC* TFs have already been functionally characterized in model plants. Zhong et al. (2012) identified two *B. napus* NAC TFs (BnNAC2 and BnNAC5) and reported their role in negative regulation of high salinity and osmotic stress tolerance. *BnNAC19* and *BnNAC82* have been shown to induce a hypersensitive response and cell death under stress due to ROS accumulation (Wang B. et al., 2015). Independent studies have reported similar observations in tobacco and *B. napus* protoplasts due to transient expression of *BnNAC55, BnNAC56, BnNAC87,* and *BnNAC103* (Niu et al., 2014; Niu et al., 2016; Chen et al., 2017; Yan et al., 2017).

Contrary to this transgenic rice overexpressing *SNAC3* showed enhanced tolerance to high temperature, drought, and oxidative stress caused by methyl viologen (MV) due to lower accumulation of ROS (Fang et al., 2015). These studies highlight the possible regulation of genes involved in ROS machinery by NAC TFs. *BnNAC485* exhibits a stress-induced gene expression (Ying et al., 2014). Overexpressing this gene in *B. napus* and *Arabidopsis* resulted in salt and osmotic stress tolerant transgenic plants. Under saline, osmotic, and ABA treatments, stress-responsive genes (*AtRD29A, AtRD29B,* and *AtABI5*) had higher expression in transgenic *Arabidopsis* plants compared to the wildtype suggesting possible regulation of BnNAC485 mediates abiotic stress response in an ABA-dependent manner. These studies also elucidate the overlapping nature of stress-responsive pathways.

#### bZIP TFs

bZIP TFs are major regulators of the stress response mechanism of plants due to their ability to recognize ABRE (ABA-responsive element), a cis-element commonly present in the promoter region of many stress-responsive genes. Most of the identified stress-responsive bZIPs are known to function in drought-responsive pathways. In *B. napus* 247 bZIP TFs have been identified, and their differential expression in various tissues and organs has been reported (Zhou et al., 2017). Zhao B. Y., et al (2016) reported the expression of *BnABF2* (gene encoding bZIP factor) in response to drought and salt stress. Further, overexpression of *BnABF2* in *Arabidopsis* conferred drought and salt resistance due to regulation of *RD29B, RAB18,* and *KIN2* genes under these stresses in an ABA-dependent manner. These studies suggest cross-talk of stress-responsive pathways and highlight the need of a multigenic approach for engineering stress-tolerant rapeseed plants.

### Transporters

Transporters are a class of transmembrane proteins facilitating the movement of selective molecules across plant membranes. They play a significant role in abiotic stress response as they control the traffic of ions and other biomolecules such as hormones and compatible solutes during stress to sustain vital cellular processes such as ion homeostasis, osmotic adjustment, signal transduction, and detoxification (Vishwakarma et al., 2019). Several transporter gene families (**Table 1**) have been identified and characterized in *B. napus* such as aquaporins (Yuan et al., 2017), metal transporter genes [MTGs; Zhang et al. (2018a)], ATP-binding cassette (ABC) transporter (Zhang et al., 2018b), sucrose transporters or sucrose carriers (SUT/SUCs) and Sugars Will Eventually be Exported Transporters [SWEET; Jian et al. (2016a)]. Most of these gene families are involved in response to drought, salt, low/high temperatures, heavy metal, and hormone treatments. The genome-wide identification of other transporter gene families such as NHX, HKT, and TMTs is further required in *B. napus* to understand their role in response to various abiotic stresses. However, Ford et al. (2012) identified orthologs of *AtNHX5* and *AtNHX6* using the *B. rapa* genome as the sequence of *B. napus* was not available at that time. They reported differential expression of *BnNHX6.1* (ortholog of *AtNHX5*) in response to salt stress. Transgenic *B. napus* plants overexpressing the *AtNHX1* gene have been reported to grow and carry out seed filling in the presence of high concentrations of NaCl (Zhang et al., 2001). Higher Na^+^ accumulation by the transgenics mediated by the Na^+^/H^+^ antiporter helped to mitigate the harmful effects of salt stress. The yield and seed quality of these transgenic plants were comparable to wild type. Similarly, overexpression of the *BnNHX1* gene in tobacco plants resulted in transgenic plants displaying enhanced salt tolerance (Wang et al., 2004). This finding highlights the utilization of antiporter genes for developing transgenic *B. napus* plants which can be grown on saline soils.

### Phytohormones

In *B. napus,* the genetic-engineering approach has been applied to modulate the levels of BRs, cytokinins, and ethylene for imparting abiotic stress tolerance, Ectopic expression of *AtDWF4* in *B. napus* was carried out to address the genetic basis by which BR signaling plays a role in abiotic stress response in *B. napus* (Sahni et al., 2016). The resultant transgenic plants showed enhanced not only plant biomass and seed yield but also significant tolerance to dehydration and heat stress in comparison to wild type. These findings suggest the utilization of genes involved in BR synthesis and signaling pathways for crop improvement. Future investigations are also clearly required to understand abiotic stress-responsive BR signaling pathways. Isopentenyltransferase (IPT) gene isolated from *Agrobacterium tumefaciens* is involved in cytokinin synthesis. Transgenic *B. napus* expressing the *IPT* gene under a developmental stage regulated promoter exhibited higher seed yield under rainfed and irrigated conditions as well as delayed leaf senescence (Kant et al., 2015). Another gene 1-aminocyclopropane-1-carboxylate (ACC) deaminase sourced from a bacterial strain *Pseudomonas putida* strain UW4 conferred transgenic *B. napus* plants with enhanced salt tolerance (Sergeeva et al., 2006). This gene decreases the negative implications of ethylene on plant growth and development by lowering the amount of ACC concentration, which is the immediate precursor of ethylene in plants.

Several other *B. napus* genes or genes sourced from other organisms involved in fatty acid metabolism, 5-Aminolevulinic acid (5-ALA) biosynthesis, phosphatidylinositol-specific signal transduction pathway, and flavonol biosynthesis have also been reported to impart tolerance to various abiotic stresses (**Table 3**). Utilization of stress-inducible promoters (e.g., *pRD29A*) can further enhance the stress-response and reduce the negative impacts of gene overexpression, if any. Engineering stress tolerance genes in the tissues more vulnerable to abiotic stress by using tissue specific promoters (Xu et al., 1993) is an efficient approach. The effective application of transgenic technology for imparting stress tolerance in *B. napus* can be further achieved by introducing multiple stress regulating genes at the same time. This can be attained either by gene pyramiding or utilizing multi-gene transformation vectors. In this direction, Wang, Z., et al (2018) took advantage of Gateway technology and the multiple rounds *in vivo* site-specific assembly (MISSA) method and introduced five different genes into a multi-gene transformation vector pABA-oriT. NCED3 (Nine-Cis-Epoxycarotenoid Dioxygenase 3), ABAR (ABA Receptor, magnesium-chelatase subunit chlH), CBF3 (C-repeat Binding Factor 3), LOS5 (molybdenum cofactor sulfurase, ABA3), and ICE1 (interactor of little elongation complex ELL subunit 1) were the five genes introduced. The resultant transgenics exhibited enhanced growth when compared to wildtype. Single gene effect or combinatorial effect of multiple genes rendered these transgenic plants tolerant to multiple abiotic stresses including salinity, drought, and heat.

**Table 3:** Studies with transgenic overexpression of stress responsive genes in *B. napus* with the aim of imparting abiotic stress tolerance.

Gene	Source of gene	Stress responsiveness in transgenics	Stress responsive physiological traits	Reference
***Genes involved in stress sensing and signalling***				
***MAPK1*** (MAP kinase)	*B. napus*	Enhanced drought tolerance	Enhanced growth and better root system under drought stress	Weng et al. (2014)
***ThIPK2*** (Inositolphosphate kinase)	*Thellungiella halophila*	Enhanced tolerance	Less wilting under drought, enhanced sodium concentrations in roots, increased seed germination, growth rate and biomass accumulation under salt stress	Zhu et al. (2009)
***Transcription Factors***				
***CBF-1,-2,-3*** (C repeat/DRE bindinG factors	*B. napus*	Enhanced freezing tolerance	N/A	Jaglo et al. (2001)
***DREB2C*** (DREB TF)	*Arabidopsis*	Enhanced salt tolerance	Higher accumulation of sodium, water, better growth and lower RWC	Song et al. (2014)
***TTG2*** (WRKY TF)	*B. napus*	Hypersensitivity to salt stress	Reduced biomass, altered leaf morphology under salt stress	Li, Q., et al (2015)
***NAC485*** (NAC TF)	*B. napus*	Enhanced osmotic and high salinity tolerance	Enhanced biomass accumulation	Ying et al. (2014)
***Transporters***				
***NHX1*** (Antiporter)	*Arabidopsis*	Enhanced salt stress tolerance	Higher sodium accumulation and enhanced survival under salt stress	Zhang et al. (2001)
***Genes involved in phytohormone signaling***				
***DWF4*** (BR biosynthesis gene)	*Arabidopsis*	Enhanced tolerance	Better seed yield, higher seed-oil content, higher root biomass and root length	Sahni et al. (2016)
***SIP 1-1*** (Trihelix TF)	*B. napus*	Enhanced osmotic and ABA tolerance, no difference under salt stress	Improved seed germination under osmotic, salt and ABA treatment, seedling survival enhanced under osmotic and ABA but not salt stress	Luo et al. (2017)
***ABI1*** (Protein phosphatase 2C)	*Arabidopsis*	Reduced drought tolerance	Reduced water retention capacity	Babula-Skowrońska et al. (2015)
***ACC-deaminase*** (1-aminocyclopropane-1-carboxylate)	*B. napus*	Enhanced salt tolerance	Higher sodium content in leaf tissues	Sergeeva et al. (2006)
***IPT*** (Cytokinin biosynthesis gene)	*Agrobacterium*	Enhanced drought tolerance	Delayed leaf senescence, enhanced seed yield, green canopy	Kant et al. (2015)
***Other stress-responsive genes***				
***YHem1*** (Involved in 5ALA synthesis)	*Yeast*	Enhanced salt stress tolerance	Higher chlorophyll accumulation, photosynthetic rate, better yield under heat stress	Sun et al. (2015)
***cyp11A1*** (cytochrome p450)	*B. napus*	Enhanced short term heat stress tolerance	Higher chlorophyll A and carotenoids	Sakhno et al. (2014)
***Ptdlns-PLC2*** (Phosphatidylinositol-specific phospholipase C)	*B. napus*	Enhance drought tolerance	Early transition to reproduction, increased photosynthetic rate	Georges et al. (2009)
***IrrE*** (Regulatory protein)	*Deinococcus radiodurans*	Enhanced salt tolerance	Enhanced growth and yield	Pan et al. (2009)
***SAD*** (Stearoylacyl carrier protein desaturase)	*Sapium sebiferum*	Enhanced freezing tolerance	Increased PUFAs content	Peng et al. (2018)
***DFR*** (Dihydroflavanol 4-reductase)	*Arabidopsis*	Enhance salt tolerance	Higher anthocyanin accumulation	Kim et al. (2017)
***Choline oxidase***	*B. napus*	No impact		Huang et al. (2000)

## Non-Coding RNAs and Multiple Abiotic Stresses

Abiotic stresses trigger transcriptional, post-transcriptional, and translational regulation of the expression of stress-responsive genes (Contreras-Cubas et al., 2012; Jeknić et al., 2014). Emerging evidence has shown that non-coding RNAs (ncRNAs) also participate in the modulation of stress-responsive gene expression. However, stress-responsive regulatory networks concerning ncRNAs are poorly understood, and unravelling such mechanisms is further a convoluted yet necessary task.

The ncRNAs are functional RNAs that do not encode or have a lower potential to encode proteins. These are a diverse group of RNA molecules classified mostly based on their location, length, genomic origin, or mode of action. A series of functionally important non-coding RNAs have been identified including the canonical ncRNAs such as transfer RNAs (tRNAs) and ribosomal RNAs (rRNAs); and regulatory RNAs, such as micro- RNAs (miRNAs), long non-coding RNAs (lncRNAs), circular RNAs (circRNAs), and others (Shin and Shin, 2016). In this section, we will review the role of ncRNAs in abiotic stress response and tolerance mechanisms.

### miRNAs and Abiotic Stress

miRNAs are short, single-stranded, 20–24 nucleotide long RNA molecules that control the expression and accumulation of target mRNAs. They indirectly modulate various biological processes such as organ development (Chen, 2004; Xie et al., 2015), phase transition (Hong and Jackson, 2015), stress response (Hackenberg et al., 2015; Zhao J. et al., 2016; Megha et al., 2018b), and several other plant regulatory pathways (Curaba et al., 2014). miRNAs originate from primary miRNA (pri-miRNA), which arise from the transcription of nuclear-encoded miRNA (MIR) genes, usually by DNA dependent RNA Polymerase II or RNA polymerase III (Voinnet, 2009; Wang J. et al., 2019). Interestingly, MIR genes are differentially regulated in response to abiotic stress in a species or family-dependent manner. Thus, the differential regulation of a miRNA is also species-specific, and so is the regulation of their target mRNAs. For instance, miR166 is reported to upregulate in wheat, whereas its expression is downregulated in rice under drought conditions (Shriram et al., 2016).

The miRNA dependent regulation of the target mRNA gene expression occurs either at the transcriptional level by site-specific methylation or at the translational level by the degradation of mRNA, inhibition of translation or RNA deadenylation (Wang J. et al., 2019). miRNAs regulate their target genes in a “Universal Reverse Manner”. This means that if a miRNA is upregulated, then its target genes are downregulated and vice versa. The activity of miRNAs in the stress-responsive regulatory networks is not well understood, and this task becomes more challenging as the response of a particular miRNA to the same abiotic stress is species-, developmental stage-, or tissue-specific (Sun X. et al., 2018). In addition, unravelling the exact mechanism of the regulation of stress response at miRNA level becomes difficult due to the regulation of several genes by one miRNA or multiple miRNAs regulating the expression of a single gene (Basso et al., 2019).

Recent investigations have revealed that miRNAs responsive to multiple abiotic stresses (Shriram et al., 2016; Basso et al., 2019). A series of stress-responsive miRNAs have been identified in *Arabidopsis* and other crop plants in response to drought, salinity, cold, heat, heavy metal toxicity, and nutrient deficiencies. Therefore, we will focus only on the differentially regulated miRNAs in *B. napus* in response to abiotic stress. In *B. napus*, the identification and expression analysis of miRNAs has been reported in response to cold temperature (Megha et al., 2018a), high concentrations of cadmium (Huang et al., 2010; Zhou et al., 2012; Jian et al., 2018) and also during early stages of seed germination under salt and drought stress (Jian et al., 2016b). These studies highlight the role of *B. napus* miRNAs in mediating developmental processes and abiotic stress response.

A total of 129 differentially regulated miRNA were identified in canola under cold stress and out of which only 25 were known (Megha et al., 2018a). Cold Stress (CS) responsive miRNAs such as miR169, miR319, miR396, which were reported in *Arabidopsis*, sugarcane, and soybean, were not observed in the case of *B. napus.* Also, cold stress responsive miRNAs did not show any consistent expression patterns with the miRNAs reported in other crops. The target genes of the CS responsive miRNAs were associated with different abiotic stresses such as heat, salt, and drought. Salt- and drought-responsive miRNAs during early-stage seed germination were identified by Jian et al. (2016b). Downregulation of miR156, miR169, miR860, miR399, miR71, and miR395 and upregulation of miR172 in response to drought was reported. MiR393 and miR399 showed an opposite regulation in response to salt stress. The identified miRNAs targeted disease resistance protein (DIRP), drought-responsive family protein (DRRP), early responsive to dehydration stress protein (ERD), stress-responsive alpha-beta barrel domain protein (SRAP), and salt tolerance homolog 2 (STH2), highlighting the regulation of stress-responsive genes by miRNAs.

In response to cadmium stress 44 known miRNAs (belonging to 27 families) and 103 novel miRNAs were identified in *B. napus*. Genes involved in transcription factor regulation, biotic stress response, ion homeostasis, and secondary metabolism were identified as corresponding target genes (Jian et al., 2018). Several Cd stress-responsive miRNAs have been reported and related regulatory mechanisms have also been discussed by Huang et al. (2010) and Zhou et al. (2012). miR395 and corresponding targets (BnSultr2;1, BnAPS3, and BnAPS4) as key regulators of cadmium stress were reported by Huang et al., 2010. Overexpression of miR395 in *B. napus* results in enhanced cadmium stress tolerance (Zhang et al., 2013). Transgenics exhibited a lower degree of Cd stress-induced oxidative damage, higher levels of biomass, and lower rates of Cd translocation from roots to leaves. The transgenics had higher expression of genes involved in heavy metal-tolerance such as *BnPCS1, BnHO1,* and *Sultr1;1*.

### lncRNAs and Abiotic Stress

lncRNAs are ncRNA molecules that range from >200 nucleotides to >10 kb in length. In plants, the transcription of lncRNAs is carried out by RNA polymerase II, III, IV/V (Wierzbicki, 2012). The biogenesis of plant lncRNAs is not thoroughly understood. Among the hypotheses, lncRNAs can originate from duplication of existing lncRNAs, the decay of protein sequences, or transposable elements (Hou et al., 2019). lncRNAs have been classified based on their genomic location as (1) natural antisense transcripts (NATs): transcribed from the opposite strand of a gene transcript, (2) sense lncRNAs: transcribed from the same strand of a gene transcript, (3) intronic lncRNAs: transcribed from the introns and (4) intergenic lncRNAs (lincRNAs): transcribed from intergenic regions (Ma et al., 2013).

In *B. napus*, oil biosynthesis (Shen et al., 2018) and cadmium stress-responsive (Feng et al., 2016) lncRNAs have been identified suggesting their role in fatty acid metabolism and cadmium stress. A number of studies have reported lncRNAs as differentially expressed under various abiotic stresses in other crop plants (**Table 4**). These lncRNAs are not highly conserved and show a species-specific expression, and thus finding common lncRNAs across species is less probable. For instance, out of 664 drought-responsive lncRNAs identified in maize, only 126 were known, and 538 were novel (Zhang W. et al., 2014). Similarly, 41.9% of the differentially regulated lncRNAs in response to salt and boron stress were species-specific in a hyper-arid maize variety (Huanca-Mamani et al., 2018). lncRNAs also show higher tissue and developmental stage-specific expression than protein-coding genes in response to abiotic stresses. Most lncRNAs were reported to be drought-responsive during the reproductive stage in maize (Pang et al., 2019).

**Table 4:** Abiotic stress responsive *lncRNAs* reported in crop plants.

Crop	Stress	Reference
***B. napus***	Cadmium	Feng et al. (2016)
**Maize**	Drought	Zhang, W., et al (2014)
	Salt and boron	Huanca-Mamani et al. (2018)
	Drought during reproductive stage	Pang et al. (2019)
**Rice**	Cadmium	Chen et al. (2018)
	Cadmium stress in roots	He et al. (2015)
**Wheat**	Drought	Muthusamy et al. (2015)
	Cold stress at reproductive stage	Diaz et al. (2019)
	Heat	Xin et al. (2011)
**Barley (Wild Tibetan)**	Drought	Qiu et al. (2019)
**Cotton**	Drought	Lu et al. (2016)
	Salt	Deng et al. (2018)
**Chinese cabbage**	Heat	Wang, R., et al (2015)
	Cold/heat	Song et al. (2016)
**Tomato**	Chilling	Wang, Y., et al (2018)
**Grapewine**	Cold	Wang, P., et al (2019)
***Medicago truncatula***	Drought, osmotic, salt	Wang, T.-Z., et al (2015)
**Cassava**	Cold and/or Drought	Li, S. X., et al (2017)
	Drought	Xiao et al. (2019)
**Foxtail millet**	Drought	Qi et al. (2013)

The number of lncRNAs responsive to certain stress also varies across species. In rice, 1434, cadmium stress-responsive lncRNAs are reported as compared to 301 in *B. napus* (Feng et al., 2016; Chen et al., 2018). This difference can also arise due to differences in methods applied for screening and identifying lncRNAs. 1,832 lncRNAs responsive to drought, cold, salinity, and ABA were reported in *Arabidopsis,* but the method used only detected lincRNAs (Liu et al., 2012). However, another study in the *Medicago truncatula* reported 5,634 lncRNAs responsive to drought by employing a method that can identify all types of lncRNAs (Wang T.-Z. et al., 2015).

lncRNAs acting as miRNA precursors have also been reported in response to various abiotic stresses. In *B. napus*, four lncRNAs act as precursors of miR824, miR167d, miR156d, and 156e in response to cadmium stress (Feng et al., 2016). In Tibetan wild barley out of 535 drought-responsive lncRNAs, 41 are putative miRNA precursors (Qiu et al., 2019). Similarly, in grapevine, 31 cold-responsive lncRNAs were potential precursors for 34 miRNAs where some of the miRNAs had multiple lncRNAs as precursors (Wang P. et al., 2019). Some studies have also reported lncNATs functioning as precursors of siRNAs under abiotic stress. lncNATs then participate in gene silencing of stress-responsive mRNAs through siRNAs. For instance, 34 lncNATS served as putative precursors of siRNAs, potentially targeting 37 cadmium stress-responsive mRNAs in *B. napus* (Feng et al., 2016). A number of lncNATs out of the 153 cold and/or drought-responsive lncNATs identified in cassava led to the formation of siRNAs (Li S. X. et al., 2017).

Target mimicry is another important mechanism by which lncRNAs regulate gene expression. miRNAs controlling the expression of stress-responsive mRNAs are blocked by binding of decoy lncRNAs. Number of lncRNAs have been reported to act as target mimics under various stresses for, e.g., 16 in cassava under cold and/drought stress (Li S. X. et al., 2017), 186 in wheat under cold stress (Diaz et al., 2019), 40 in Chinese cabbage under heat stress (Wang A. et al., 2019), and 3,560 in Barley under drought stress (Qiu et al., 2019). The number of target mimics and the number of miRNAs is not the same, indicating a complex network of cross-talk between miRNA and lncRNA. The functional validation of three cadmium stress-responsive lncRNAs which were predicted as target mimics identified an association between those lncRNAs and cadmium responsive genes such as natural resistance-associated macrophage protein 1 (Nramp1) type metal transporter and a Cu/Zn superoxide dismutase which was a component of the oxidative response machinery and metal transporters (Feng et al., 2016).

Cross-talk between lncRNA and miRNAs is suggested to impact the expression of various transcription factors with an important role in abiotic stress response. MiRNA-lncRNA co-expression networks in Chinese cabbage identified several transcription factors such as DREB2A, HSFs, ARFs, and HSPs under heat stress (Wang A. et al., 2019). Similarly, under drought and/or cold stress in cassava, 164 lincRNAs targeted NAC TFs, which play a role in drought tolerance, and 169 lincRNAs targeted NF-Y TFs which play a role in abiotic stresses (Li S. X. et al., 2017). These findings suggest an intricate mechanism by which lncRNAs might regulate stress response that needs experimental validation.

Although lncRNAs have been reported to be stress-responsive, their functional characterization is mostly lacking. In *Arabidopsis*, however, a drought, salt, and ABA-responsive lncRNA have been characterized. The DROUGHT INDUCED lncRNA (DRIR), which when overexpressed in *Arabidopsis*, imparted salt and drought tolerance and was reported to be more sensitive to ABA treatments. The gene expression in these overexpression lines also elucidated that this lncRNA acts as a positive regulator of abiotic stress response in *Arabidopsis* (Qin et al., 2017). Similar research is essential for other identified lncRNAs to facilitate the identification of candidates, which can impart multiple abiotic stress tolerance.

### circRNAs and Abiotic Stress

circRNAs are a class of newly characterized endogenous ncRNAs which are 100 nt to 4 kb in length. circRNAs were reported for the first time in the year 1976 as single-stranded circular RNA molecules in plant viroids (Sanger et al., 1976). These circular RNA molecules were considered as splicing errors for several decades due to their lower level of expression and difficulties involved in identification (Cocquerelle et al., 1993). However, with the advancement in sequencing technologies, genome-wide investigations have led to the discovery of thousands of circRNAs in bacteria (Danan et al., 2011), fungi (Wang et al., 2014), animals (Memczak et al., 2013), and human cells (Salzman et al., 2012). In plants, the first report of circRNA characterization is in *Arabidopsis* (Wang et al., 2014). They are highly conserved in eukaryotes and are involved in various biological processes (Ye et al., 2015). The biogenesis of circRNAs is unclear in plants. In animals, circRNAs are suggested to act as miRNA sponges and hence interfere with gene silencing mediated by miRNAs (Memczak et al., 2013). In plants, there is no experimental evidence so far to validate this mode of action; however, there are some studies that predict a potential role of plant circRNAs as miRNA sponges (Conn et al., 2017).

Abiotic stress regulation by circRNAs has not been extensively studied in plants. Few studies have reported stress-responsive expression of circRNAs in crop plants (Zhao et al., 2019). However, such research is missing in *B. napus*. In *Arabidopsis*, circRNAs responsive to drought (Zhang P. et al., 2019) and heat (Pan T. et al., 2018) stresses have been identified. Pan T., et al (2018) identified 1,583 heat-responsive circRNAs in *Arabidopsis* and highlighted that heat stress promotes the expression, abundancy, and exon circularization of circRNAs. Studies in other crops has also uncovered circRNAs responsive to phosphate starvation [rice, Ye et al. (2015)], drought [maize, Zhang, P., et al (2019); wheat, Wang, Y., et al (2017)] and cold [tomato, Zuo et al. (2016); grape, Gao et al. (2019)]. These studies suggest that circRNAs might regulate abiotic stress response in plants by modulating the stress-responsive gene expression. Though, the mechanism is not very clear, but it can be speculated from current research that either circRNAs act as miRNA sponges or they inhibit the biogenesis of sRNAs (Zhang P. et al., 2019) thus protecting the stress responsive transcripts from gene silencing.

Experimental evidence has validated the molecular mechanisms involved in circRNA-mediated stress response in the case of two circRNAs. CircGORK (Guard cell outward-rectifying K+-channel) was overexpressed in *Arabidopsis* (Zhang P. et al., 2019) for functional characterization. GORK gene is involved in water stress response and regulation of ABA-signaling. CircGORK overexpression lines suggested a positive regulation of drought tolerance by upregulation of several ABA-responsive genes. Similarly, a cold-responsive grape circRNA Vv-circATS1 was overexpressed in *Arabidopsis* (Gao et al., 2019). The overexpression of this circRNA resulted in enhanced cold tolerance, whereas its linear counterpart failed to show a similar impact. These reports provide functional tools and a framework for further characterization of circRNAs and their role in stress response regulation. circRNAs are also suggested to be utilized as molecular markers for breeding stress-tolerant varieties. Therefore, new research is warranted for the identification of candidate circRNAs that can be explored further for developing multiple stress-tolerant crop varieties.

In recent years, the role of several stress-responsive genes and ncRNAs has been extensively studied in plant's abiotic stress responses. Tremendous work has been carried out in developing databases and bioinformatics tools for stress-responsive gene identification and profiling. A comprehensive and more in-depth understanding of their mode of action, differential regulation patterns, identification of target genes, and ncRNA mediated gene regulatory machinery is necessary for *B. napus*. Investigations focusing on employing advanced tools of synthetic biology in cooperation with genetic engineering to develop an effective strategy for designing multiple abiotic stress-tolerant varieties in *B. napus* as well as other economically important crops are required.

## Biotechnological Approaches and Synthetic Biology for Developing Climate Change Resilient *B. Napus* Varieties

Stress tolerance is a polygenic response due to the complex nature of networks involved in abiotic stress sensing, signal transduction, and expression of stress-responsive genes in plants. Gene transformation technologies have been successfully applied for imparting different stress tolerances in plants. However, this genetically complex mechanism of an abiotic stress response makes the task of engineering crops tolerant to multiple abiotic stresses extremely challenging.

### Genome Editing Techniques

Genome editing is an efficient approach for crop improvement either by loss of gene function, the gain of gene function, or a multiplex genome editing approach. Several different strategies have been developed for genome editing in plants due to the advent of engineered or designer nucleases. These nucleases can introduce double-stranded breaks (DSBs) at specific sites on the genome. These DSBs are then repaired either by non-homologous end joining (NHEJ) or homology-directed repair (HDR). NHEJ is error-prone, whereas HDR results in precise insertion, deletion, or substitution events (Sedeek et al., 2019). The nucleases that found application in efficient genome editing in plants are ZFNs (Zinc finger nucleases), TALENs (Transcription activator-like effector nucleases), and CRISPR-Cas9. Each class of nucleases has its advantages and disadvantages. However, CRISPR-Cas9 (Clustered regularly interspaced short palindromic repeats-Cas9) due to its simplicity and precision was quickly established as the preferred gene-editing technique.

The discovery of CRISPR and its subsequent adaptation to CRISPR/Cas9 technology was a revolutionary step in genome editing. Three CRISPR systems have been distinguished (types I–III). However, it is type II, which is successfully modified to the currently renowned CRISPR/Cas9 technology (Hsu et al., 2014). This technology involves the inclusion of Cas9 nuclease, which binds to a constructed single guide RNA (sgRNA) (Doudna and Charpentier, 2014). sgRNA consists of an RNA-duplex and a guide sequence, this sequence determining the target DNA to which the sgRNA will bind before the associated Cas9 induces a double-stranded break (DSB). Although this approach is simple and easy, there is a limitation of offtargets. To reduce off-targeting by 50–1,000 folds, the use of mutant Cas9 known as DNA nickase has been reported (Cong et al., 2013; Ran et al., 2013). Other approaches include the fusion of fok1 nuclease with catalytically inactive cas9 protein (Tsai et al., 2014) and efficient designing of sgRNAs. The advancement in CRISPR/Cas9 system has led to the selection and screening of the transgenic plants in a way that only the plants with the desired mutations and lose of the transgene are screened for (Bao et al., 2019). This provides a possible solution for the development of the non-GM crop, which can bypass the strict biosafety regulations required for genetically modified crops.

In *B. napus*, a few studies have utilized CRISPR/Cas9 system for editing genes associated with plant/pod development (ALCATRAZ, GA1-3, FRUITFULL, DA1, DA2, CLAVATA, and SPL3), fatty acid synthesis (BnFAD2), and biotic stress response (BnWRKY11and -70) (Sun Q. et al., 2018). The application of CRISPR-Cas9 for climate-resilient transgenic production is yet to be reported in canola (**Table 5**).

**Table 5:** Application of CRISPR-Cas9 for improving abiotic stress tolerance in crop plants.

Target gene	Crop	Trait	Reference
***SAPK2***	Rice	Increased sensitivity to drought stress and ROS	Lou et al. (2017)
***ARGOS8***	Maize	Drought stress tolerant and enhanced grain yield under	Shi et al. (2017)
***SlMAPK3***	Tomato	Drought tolerance	Wang, L., et al (2017)

### Gene Delivery Tools

The workflow for generating a genetically engineered plant entails the delivery of DNA to plants, followed by transformants selection and regeneration of the genetically modified progeny. Delivery of DNA into plant cells can be achieved either by direct DNA delivery methods or *Agrobacterium-*mediated transformation. Direct DNA transfer methods such as PEG-mediated DNA uptake, electroporation, microinjection, and microprojectile bombardment have been explored for *B. napus* (Bergman and Glimelius, 1993; Jones-Villeneuve et al., 1995; Poulsen, 1996). Isolation of protoplasts and difficulties in regeneration of viable plants due to increased risk of somaclonal variations make the application of PEG mediated DNA uptake and electroporation limited. Microinjection for delivering genes to microspores as well as microprojectile bombardment has been successfully carried out in *B. napus* (Jones-Villeneuve et al., 1995). However, particle bombardment has low precision and relies on high-pressure delivery of DNA-coated gold particles by physical disruption of the cell wall, which can lead to multiple gene copies, DNA fragmentation, or integration of vector backbone.

The most common method for generating genetically modified *B. napus* is *Agrobacterium*-mediated transformation (Zhang et al., 2005; Bhalla and Singh, 2008). This method has also been successfully applied to other Brassica species (Bhalla and Smith, 1998; Smith and Bhalla, 1998; Bhalla and De Weerd, 1999). There are many success stories in the generation of GM canola with insect resistance, herbicide tolerance, or both as well as enhanced production of omega-3 fatty acids. These GM canola varieties are grown successfully, and the agriculture industry has reaped rewards of higher yield, better nutritional quality oil, and other agro-economic benefits by the adoption of GM canola technology. Transgenic research focusing on the development of abiotic stress tolerant *B. napus* also successfully utilizes *Agrobacterium*-mediated transformation techniques. However, drawbacks of this approach include the random nature of the gene insertion, possibility of disrupting functional genes, public concerns over genetically modified organisms (GMOs), failure to make use of the native genetic repertoire of the plant and subjections to GMO regulatory regulations. Also, there is a question of recalcitrant commercial canola varieties that require more efficient transformation techniques (Zhang and Bhalla, 2004).

Approaches challenging how GM crops are developed, regulated, and labeled are also required. This can be achieved by controlling biomolecule delivery to plants in an unassisted and accurate manner. In this direction, the application of nanotechnology for DNA delivery provides a potential solution (Landry and Mitter, 2019). Materials having at least one dimension >100 nm are defined as nanoparticles. When compared with the exclusion limit of a typical cell wall, which is 500 nm, these nanoparticles can act as efficient carriers of biomolecules into the plant cells. So far, nanoparticles such as mesoporous silica (few 100 nm) with a gene gun (Torney et al., 2007) and polyethyleneimine-coated Fe_3_O_4_ magnetic nanoparticles (MNP) using magnetic force (Zhao et al., 2017) have been investigated as nanocarriers of DNA in plant cells. Use of magnetic nanoparticles for transforming pollen and then pollinating plants with this magnetofected pollen resulted in the successful generation of transgenic cotton. The DNA delivered by this method was stably integrated into the genome and inherited in the successive generations (Zhao et al., 2017). Several other nanoparticles, such as carbon nanotubes (Demirer et al., 2019; Kwak et al., 2019), DNA nanostructures, and DNA origami (Zhang H. et al., 2019) have been successfully investigated for unassisted delivery of exogenous DNA. Recent advances in genome editing tools and pioneering research involving nanotechnology for transformation provides precise, unique, and efficient tools for generating GM crops. The integration of these technologies will shorten the time required for developing GM crop varieties and might assist in bypassing the GM regulatory purview, thus resulting in higher consumer acceptance of GM crops.

### Synthetic Biology as an Emerging Approach

Plant synthetic biology, a rapidly emerging field that aims to combine plant biology with engineering principles (Kassaw et al., 2018). Initially, it was applied to bacterial systems, but now it has expanded to eukaryotic systems, including plants. Designing and construction of synthetic regulatory systems exhibiting desirable and predictable behavior using either a top to bottom or bottom-up design approach is a very useful tool which can be applied for developing plant varieties with novel traits.

For developing sentinel plants exhibiting desirable traits, the efficient designing of genetic circuits is a pre-requisite. The designing and assembly of genetic circuits can be carried out using various software such as Bio-CAD for designing (Nielsen et al., 2016) and Cell Modeller (Dupuy et al., 2007) for assembly of genetic parts. The orthogonal genetic components should have the ability to function independently of the endogenous regulatory mechanism of the plant (Morey et al., 2012; Chen et al., 2013). Also, the ability to externally control the activation of the genetic circuits can help us switch off/on the desirable trait as and when required. The incorporation of regulatory components such as terminators and insulators provide better control of the synthetic genetic circuit (Chen et al., 2013). However, plant genetic functions are complex and regulated by various environmental cues such as light, temperature, photoperiod, which in turn impact the synthetic gene circuit control. These factors, along with the gene regulatory information available from different omics studies, should be incorporated while developing sentinel plants.

Plant biosensors have been developed capable of sensing 2,4,6-trinitrotolune (TNT) *via* a two-component synthetic gene circuit (Antunes et al., 2011). First component acts as the transmembrane signal activator upon exposure to TNT. It then triggers the second component by activating the synthetic promoter. The promoter controls the activity of genes which inhibit chlorophyll synthesis and initiates its degradation. *Arabidopsis* and lettuce protoplasts were modified to signal for bacterial contamination efficiently *via* a gene circuit controlled by a positive autoregulatory transcriptional feedback loop (Czarnecka et al., 2012). This emerging technology has the potential to be applied broadly, with the development of sentinel plants that detect specific or multiple abiotic stresses such as heat, salinity, and drought in the field and initiates a gene regulatory network for imparting stress tolerance.

## Conclusion

The agronomic importance of *B. napus* in global agriculture is a vital factor driving the necessity for innovative tools to combat multiple stresses in the current climatic scenario. Will biotechnology and particularly synthetic biology be the essential pieces to this puzzle are still needed to be developed fully, but the early indications do present a promising picture. The availability of the *B. napus* genome has led to the identification and characterization of various stress-responsive gene families such as HSFs, WRKY TFs, bZIP TFs, metal transporters, and so on. However, its in-depth analysis to untangle the complex stress-responsive networks and their cross-talks require further research. Identification of putative multiple abiotic stress regulatory genes or regulatory sequences and their efficient manipulation can lead to the development of the ultimate climate change resilient *B. napus* varieties. Several studies have reported the application of conventional genetic engineering for imparting abiotic stress tolerance in *B. napus* varieties. Overexpression of transcription factors, transporters, and genes involved in phytohormone biosynthesis, miRNAs, and other stress-responsive genes have been reported to be promising approaches for combating multiple abiotic stresses. Multi-gene co-expression for conferring stress tolerance in *B. napus,* as reported by Wang, Z., et al (2018), highlights the need for similar future research. Application of CRISPR/Cas9 editing technology for developing multiple abiotic stress resistance in *B. napus* is also required. Further optimization of transformation protocols by multiplex genetic engineering, application of nanoparticles for gene delivery, and synthetic biology for developing gene circuits for rapid signaling and efficient initiation of abiotic stress responses are opening new opportunities in this direction. The integration of innovative tools into the current genetic engineering programs will provide new prospects for the rational design of multiple stress-tolerant traits in *B. napus*.

## Author Contributions

PB and MS conceived the concept and the idea and supervised the project. NL and DJ collected the literature and compiled the information and wrote the article. MS and PB extensively edited the article.

## Conflict of Interest

The authors declare that the research was conducted in the absence of any commercial or financial relationships that could be construed as a potential conflict of interest.
